# Metabolic changes after growth hormone treatment in children with growth hormone deficiency and small for gestational age: LG observational study

**DOI:** 10.3389/fendo.2025.1661018

**Published:** 2025-11-19

**Authors:** Sujin Kim, Seo Jung Kim, Kyoung Won Cho, Youngha Choi, Kyungchul Song, Eun Byoul Lee, Hyun Wook Chae, Junghwan Suh

**Affiliations:** 1 Department of Pediatrics, Severance Children’s Hospital, Yonsei University College of Medicine, Seoul, Republic of Korea; 2 Department of Pediatrics, Gangnam Severance Hospital, Yonsei University College of Medicine, Seoul, Republic of Korea; 3 Department of Pediatrics, Yongin Severance Hospital, Yonsei University College of Medicine, Yongin, Republic of Korea

**Keywords:** glucose metabolism, growth hormone, lipid profile, metabolic health, short stature in children, linear growth, retrospective study

## Abstract

**Purpose:**

Children with growth hormone deficiency (GHD) and small for gestational age (SGA) have major growth and metabolic issues. Growth hormone (GH) treatment promotes linear growth and improves metabolic parameters. However, the long-term metabolic effects of this treatment remain unclear. This study aimed to investigate metabolic changes after GH treatment in children with GHD and SGA.

**Methods:**

The study population included 1,788 patients: 1,364 with GHD, 318 with SGA, and 106 with GHD and SGA. Data on growth and metabolic parameters, age, sex, height, weight, body mass index (BMI), and various biochemical markers before and after at least one year of GH treatment were collected from the LG observational study.

**Results:**

GH treatment was initiated at 7.3 ± 2.8 years (GHD: 7.4 ± 2.9 years; SGA: 6.6 ± 2.5 years), with an average treatment duration of 3.8 ± 1.3 years. In the GHD and SGA patient groups, the height and weight standard deviation scores (SDS) significantly increased during treatment. BMI SDS significantly decreased in the first year in both groups; the SGA group showed improvement in years 1–5. AST levels significantly decreased over the 5-year treatment period in both groups. ALT levels significantly decreased in all follow-up years in the GHD group. In both groups, total cholesterol levels decreased and random glucose levels remained within the normal range.

**Conclusion:**

These findings demonstrate a positive effect of GH treatment on growth and metabolic parameters in children with GHD and SGA.

## 1 Introduction

Growth hormones (GH) play a crucial role in promoting linear growth. It is widely used to treat growth disorders, including growth hormone deficiency (GHD), short stature in children born small for gestational age (SGA), and other syndromic conditions (1). Beyond its essential role in linear growth, GH treatment also influences metabolic regulation by normalizing lean body mass, supporting glucose metabolism and insulin secretion (2), and improving cardiovascular lipid profiles, and potentially reducing metabolic risk factors (3).

GH serves as a lipolytic hormone that promotes energy expenditure and reduces visceral fat (4). In adults with GHD, GH treatment can improve lipid profiles by lowering total cholesterol and low-density lipoprotein (LDL) cholesterol levels (5, 6). Additionally, GH treatment has been associated with reductions in body mass index (BMI) and visceral adiposity (7). Untreated adults with GHD have an increased risk of complications, including cardiovascular and liver diseases, metabolic dysfunction-associated steatotic liver disease (MASLD), and insulin resistance (8). In pediatric patients with GHD, the metabolic effects of GH remain poorly documented, as most existing studies have primarily focused on its role in promoting linear growth. However, some retrospective studies have reported significant improvements in various components of metabolic syndrome among children and adolescents with GHD (9, 10). Furthermore, when GHD results from underlying conditions such as craniopharyngioma requiring surgical intervention, the metabolic benefits of GH treatment remain controversial (11, 12).

For children with SGA, GH treatment facilitates catch-up growth and improves the final adult height. However, patients with SGA have an elevated risk of various metabolic complications during catch-up growth. In adulthood, they may also experience increased insulin resistance, obesity, and cardiovascular diseases (13). Studies suggest that GH treatment is most effective in improving final adult height when started early, maintained for a longer duration, and administered at higher doses (14). However, given the necessity of minimizing side effects, the management of GH treatment in this population presents a unique challenge. Close monitoring of lipid and glucose metabolism indicators throughout GH treatment is strongly recommended to ensure both safety and effectiveness. Nevertheless, there is an ongoing debate regarding the potential metabolic risks and benefits associated with GH treatment. Some studies indicate that GH treatment may increase glucose and insulin levels (4), whereas others highlight its positive effects on lipid metabolism (15).

To address this knowledge gap, we aimed to clarify the effect of GH treatment on the growth and metabolic parameters of pediatric patients with GHD and SGA by analyzing data from a large observational cohort, thereby providing valuable insights into its overall benefits and risks.

## 2 Methods

### 2.1 Design and study population

**Figure 1:**
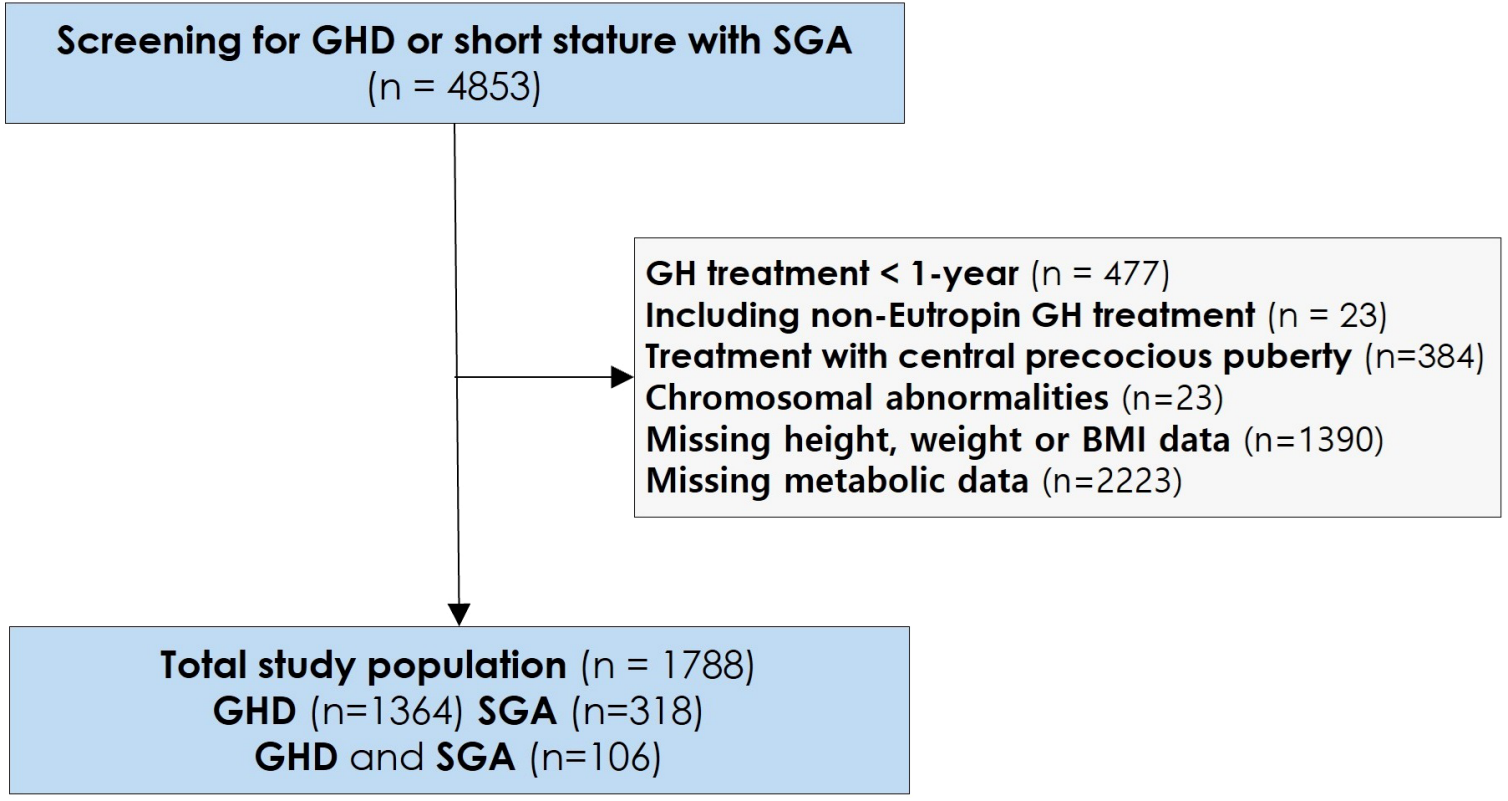
Study selection and baseline population. GH, Growth Hormone; GHD, Growth Hormone Deficiency; SGA, Small for Gestational Age; BMI, Body mass index.

This retrospective study included 1,788 patients enrolled in the LG Growth Study (LGS), a multicenter registry designed to evaluate the effectiveness and long-term safety of GH treatment. Patients received recombinant human growth hormone (rhGH) in the form of Eutropin injection, Eutropin Pen^®^, and Eutropin S-pen^®^ (all containing somatropin, LG Chem, Ltd., Seoul, Republic of Korea). The study design has been previously described in detail (16). The study population comprised 1,364 patients with GHD, 318 with SGA, and 106 with both GHD and SGA. The presence of GHD was determined based on a height below -2 standard deviations (SD) for the corresponding sex- and age-matched cohorts (17) and a GH peak response of less than 10 ng/mL in two GH stimulation tests. For individuals born SGA according to the Korean reference (18) with short stature, the criteria included a height below -2 SD and a birth weight below -2 SD from the mean for the corresponding gestational age and sex. The study participants were screened for eligibility to determine whether they had received daily GH treatment in the LGS between December 2011 and March 2024. The selection process for the study population is shown in **Figure 1**.

### 2.2 Assessments and measurements

Annual height and weight data were collected from medical records, starting from pre-treatment and continuing up to 5 years post-treatment. Standard deviation scores (SDS) were calculated based on the 2017 Korean growth charts (17). Mid-parental height, defined as the average height of both parents with 6.5 cm added for males and 6.5 cm subtracted for females, was recorded when available.

Metabolic parameters were also assessed. Aspartate aminotransferase (AST) and alanine aminotransferase (ALT) levels were used as indicators of liver function. Total cholesterol, triglyceride (TG), and uric acid levels were determined using standard enzymatic methods. Random glucose and glycosylated hemoglobin (HbA1c) levels were also quantified using standard enzymatic methods. Total cholesterol, TG, and glucose levels were measured in a non-fasting state. Data for all available parameters were collected from the LGS cohort, covering the period from pre-treatment to 5 years post-treatment.

### 2.3 Statistical methods

Statistical analyses were performed using SAS 9.4 (SAS Institute, Cary, NC). Variables are presented as mean ± SD. For paired data on auxological and metabolic parameters, either a paired *t-*test or a Wilcoxon signed-rank test was applied, depending on the data distribution. A *post-hoc* power analysis was performed using PASS 2020 based on the observed effect sizes of the primary outcomes, with a two-sided α = 0.05. Most analyses demonstrated a statistical power greater than 80%.

To analyze repeated measurements over time, generalized estimating equations (GEE) were used to evaluate the association between time and clinical variables while adjusting for age, sex, and GH dosage. The GEE model was fitted with a Gaussian family and identity link function, and an exchangeable working correlation structure was applied. To assess the robustness of these results, a sensitivity analysis was additionally performed using a linear mixed-effects model based on the repeated measures analysis of variance framework (PROC MIXED in SAS, version 9.4; SAS Institute Inc., Cary, NC, USA), which yielded consistent findings. Furthermore, Spearman correlation analysis was conducted to evaluate the relationship between changes in serum uric acid levels and anabolic markers, including BMI SDS and IGF-1, to explore potential mechanistic associations. Statistical significance was set at p ≤ 0.05.

## 3 Results

### 3.1 Baseline characteristics

This study enrolled a total of 1,788 patients with GHD or SGA, of whom 59.1% were male. Among them, 106 patients were diagnosed with both GHD and SGA. **Table 1** summarizes the baseline characteristics of the study population. The mean age at GH initiation was 7.3 ± 2.8 years. Patients with SGA began GH treatment at a younger age (6.5 ± 2.4 years) than those with GHD (7.5 ± 2.9 years, p < 0.01). The average duration of GH treatment was 3.8 ± 1.3 years. Before initiating GH treatment, the mean height SDS, weight SDS, and BMI SDS were below average, measuring -2.46 ± 0.74, -1.79 ± 1.07, and -0.44 ± 1.1, respectively. Patients with SGA had significantly lower body weight and BMI SDS than those with GHD (p < 0.01). Additionally, the mean GH peak level was 6.2 ± 2.5 ng/mL in patients with GHD and significantly higher at 16.4 ± 8.0 ng/mL in those with SGA. The mean GH treatment dose was 0.28 ± 0.06 mg/kg/week. Patients with SGA received a relatively higher dose (0.29 ± 0.06 mg/kg/week) than those with only GHD (0.27 ± 0.12 mg/kg/week, p < 0.01).

**Table 1:** Baseline characteristics of the study population.

Variables	Total (N = 1788)	GHD^a^ (N = 1364, 76.3%)	SGA^a^ (N = 318, 17.8%)	GHD–SGA^a^ (N = 106, %)	*p-*value
Male (n [%])	1056 [59.1%]	819 [60.0%]	171 [53.8%]	66 [62.26%]	0.10^†^
Age at starting GH treatment (years)	7.3 ± 2.8	7.5 ± 2.9	6.5 ± 2.4	6.8 ± 2.4	<0.01^††^
Duration of GH treatment (years)	3.8 ± 1.3	3.8 ± 1.3	3.8 ± 1.3	3.8 ± 1.2	0.45^††^
Height (cm)	112.9 ± 15.2	114.0 ± 15.5	109.2 ± 13.3	109.9 ± 14.5	<0.01^††^
Height SDS^†††^	-2.46 ± 0.74	-2.45 ± 0.74	-2.38 ± 0.63	-2.62 ± 0.76	0.02^††^
Male mid-parental height (cm)	169.9 ± 3.6	169.9 ± 3.6	170.3 ± 3.6	169.5 ± 3.7	0.58^††^
Female mid-parental height (cm)	157.5 ± 3.8	157.5 ± 4.0	157.2 ± 3.3	158.1 ± 4.0	0.35^††^
Weight (kg)	21.6 ± 8.3	22.5 ± 8.6	18.5 ± 5.7	20.0 ± 8.0	<0.01^††^
Weight SDS^†††^	-1.79 ± 1.07	-1.66 ± 1.04	-2.21 ± 0.99	-2.11 ± 1.22	<0.01^††^
BMI (kg/m^2^)	16.3 ± 2.4	16.7 ± 2.5	15.2 ± 1.7	15.9 ± 2.6	<0.01^††^
BMI SDS^†††^	-0.44 ± 1.11	-0.31 ± 1.09	-0.93 ± 1.04	-0.64 ± 1.15	<0.01^††^
GH peak in GH stimulation test (ng/mL)	6.7 ± 3.8	6.2 ± 2.5	16.4 ± 8.0	6.7 ± 2.3	<0.01^††^
Mean GH dose (mg/kg/week)	0.28 ± 0.06	0.27 ± 0.12	0.29 ± 0.06	0.30 ± 0.12	<0.01^††^

^†^p-values were calculated by Chi-square test.

^††^p-values were calculated by Kruskal-Wallis test.^†††^ The Lambda-Mu-Sigma (LMS) method is used to calculate SDS.

Data are shown as the mean ± SD.

BMI, Body Mass Index; GHD, Growth Hormone Deficiency; SGA, Small for gestational age; GH, Growth Hormone; SDS, Standard Deviation Score.

### 3.2 Changes in auxological parameters after GH treatment

**Tables 2A**-**C** present the longitudinal growth measurements. In patients with GHD, SGA, and both GHD and SGA, height and weight SDS significantly increased following GH treatment compared to baseline values (p < 0.01 at each time point from years 1 to 5 for both parameters). By the fifth year of treatment, height SDS increased to -0.83 ± 1.13 in patients with GHD, to -0.76 ± 0.96 in those with SGA, and to -1.41 ± 1.28 in those with GHD and SGA. The increase in height SDS compared to baseline was significantly greater in patients with SGA than in those with GHD in the fourth and fifth years of GH treatment (fourth year: 1.74 ± 0.72 vs. 1.63 ± 0.73, p = 0.03; fifth year: 1.86 ± 0.83 vs. 1.77 ± 0.94, p = 0.045) (**Figure 2A**). Similarly, weight SDS increased significantly in the three groups; however, the increase was consistently greater in patients with SGA than in those with GHD at each time point (p < 0.01, years 1–5) (**Figure 2B**).

**Table 2A:** Changes in auxological parameters after GH treatment in patients with GHD.

Variables	Baseline (n)	1^st^ year (n)	2^nd^ year (n)	3^rd^ year (n)	4^th^ year (n)	5^th^ year (n)	*p*-value^**^
Height SDS	-2.45 ± 0.74 (1364)	-1.65 ± 0.77^*^ (1125)	-1.26 ± 0.79^*^ (796)	-1.02 ± 0.87^*^ (547)	-0.90 ± 1.02^*^ (344)	-0.83 ± 1.13^*^ (222)	<0.01
Weight SDS	-1.66 ± 1.04 (1318)	-1.23 ± 0.97^*^ (1119)	-0.91 ± 1.01^*^ (790)	-0.72 ± 1.06^*^ (543)	-0.72 ± 1.15^*^ (344)	-0.63 ± 1.13^*^ (221)	<0.01
BMI SDS	-0.31 ± 1.09 (1317)	-0.47 ± 1.00^*^ (1119)	-0.37 ± 1.07^*^ (790)	-0.28 ± 1.07^*^ (543)	-0.38 ± 1.06 (343)	-0.31 ± 1.04 (221)	0.06

^*^p-value < 0.05 after GH treatment compared to baseline.

^**^p-value by analyzing generalized estimating equations over time.

Data are shown as the mean ± SD.

BMI, Body Mass Index; GH, Growth Hormone; GHD, Growth Hormone Deficiency; SDS, Standard Deviation Score.

**Table 2B:** Changes in auxological parameters after GH treatment in patients with SGA.

Variables	Baseline (n)	1^st^ (n)	2^nd^ year (n)	3^rd^ year (n)	4^th^ year (n)	5^th^ year (n)	*p*-value^**^
Height SDS	-2.38 ± 0.63 (318)	-1.61 ± 0.66^*^ (276)	-1.21 ± 0.72^*^ (187)	-0.90 ± 0.75^*^ (121)	-0.82 ± 1.05^*^ (80)	-0.76 ± 0.96^*^ (42)	<0.01
Weight SDS	-2.21 ± 0.99 (311)	-1.63 ± 0.93^*^ (275)	-1.31 ± 0.93^*^ (187)	-0.95 ± 1.05^*^ (120)	-0.81 ± 1.27^*^ (80)	-0.84 ± 1.33^*^ (42)	<0.01
BMI SDS	-0.93 ± 1.04 (311)	-0.98 ± 0.95^*^ (275)	-0.92 ± 0.98^*^ (187)	-0.69 ± 1.12^*^ (120)	-0.55 ± 1.30^*^ (80)	-0.64 ± 1.42^*^ (42)	<0.01

^*^p-value < 0.05 after GH treatment compared to baseline.

^**^p-value by analyzing generalized estimating equations over time.

Data are shown as the mean ± SD.

BMI, Body Mass Index; GH, Growth Hormone; SGA, Small for Gestational Age; SDS, Standard Deviation Score.

**Table 2C:** Changes in auxological parameters after GH treatment in patients with GHD–SGA

Variables	Baseline (n)	1^st^ (n)	2^nd^ year (n)	3^rd^ year (n)	4^th^ year (n)	5^th^ year (n)	*p*-value^**^
Height SDS	-2.62 ± 0.76 (106)	-1.80 ± 0.76^*^ (83)	-1.50 ± 0.88^*^ (68)	-1.25 ± 0.86^*^ (41)	-1.38 ± 0.94^*^ (27)	-1.41 ± 1.28^*^ (15)	<0.01
Weight SDS	-2.11 ± 1.22 (105)	-1.62 ± 1.12^*^ (83)	-1.37 ± 1.12^*^ (68)	-1.19 ± 1.22^*^ (41)	-1.22 ± 1.11^*^ (26)	-1.34 ± 1.04^*^ (15)	<0.01
BMI SDS	-0.64 ± 1.15 (105)	-0.79 ± 1.12^*^ (83)	-0.76 ± 1.08 (68)	-0.77 ± 1.3 (41)	-0.72 ± 1.2 (26)	-0.85 ± 1.08 (15)	0.58

^*^p-value < 0.05 after GH treatment compared to baseline.

^**^p-value by analyzing generalized estimating equations over time.

Data are shown as the mean ± SD.

BMI, Body Mass Index; GH, Growth Hormone; SGA, Small for Gestational Age; SDS, Standard Deviation Score.

**Figure 2:**
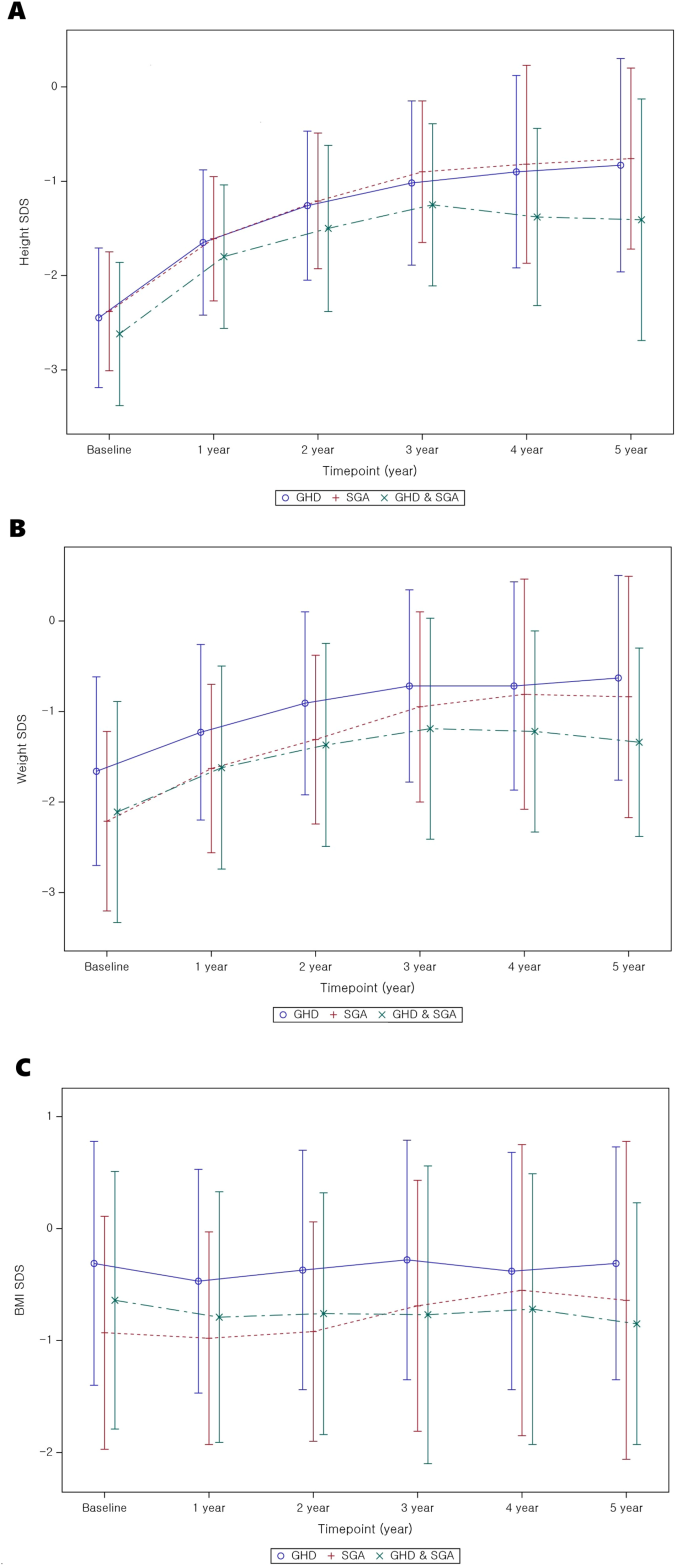
Auxological parameter changes from baseline to 5 years after GH treatment. **(A)** Height SDS; **(B)** weight SDS; **(C)** BMI SDS. GH, Growth Hormone; GHD, Growth Hormone Deficiency; SGA, Small for Gestational Age; SDS, Standard Deviation Score; BMI, Body mass index.

Additionally, the BMI SDS exhibited significant differences between the GHD and SGA groups. In patients with GHD, BMI decreased during the first year of GH treatment but increased in the second and third years. A similar pattern was observed in patients with SGA, where BMI decreased in the first year of treatment and slightly increased by the third year (**Figure 2C**). GEE analysis over time indicated a statistically significant overall increase in BMI SDS in the SGA group (p < 0.01).

### 3.3 Changes in metabolic parameters after GH treatment

**Figure 3:**
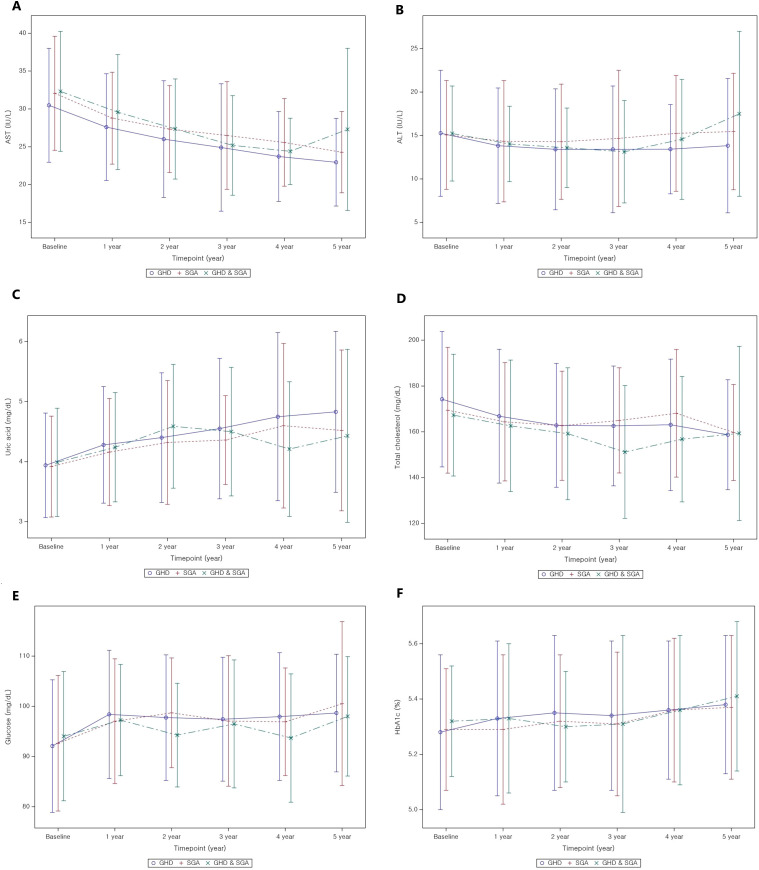
Metabolic parameter changes from baseline to 5 years after GH treatment. **(A)** AST; **(B)** ALT; **(C)** uric acid; **(D)** total cholesterol; **(E)** glucose; **(F)** HbA1c. GH, Growth Hormone; GHD, Growth Hormone Deficiency; SGA, Small for Gestational Age; AST, Aspartate Aminotransferase; ALT, Alanine Aminotransferase; HbA1c, Glycosylated hemoglobin.

**Tables 3A, B** present the longitudinal changes in metabolic parameters throughout GH treatment. In the GHD group, both AST and ALT levels significantly decreased to within the normal range from the first to the fifth year of GH treatment compared with the baseline levels (**Figure 3A, B**). GEE analysis confirmed a significant reduction in the AST and ALT levels during the treatment period (p < 0.01). Additionally, uric acid levels exhibited a statistically significant increase (p < 0.01), progressively increasing throughout the treatment duration (**Figure 3C**). Serum uric acid levels demonstrated a consistent positive association with BMI SDS at years 1–3 and with IGF-1 SDS across all time points in the GHD group (**Supplementary Tables 1**-**3**).

**Table 3A:** Changes in metabolic parameters after GH treatment in patients with GHD.

Variables	Baseline (n)	1^st^ year (n)	2^nd^ year (n)	3^rd^ year (n)	4^th^ year (n)	5^th^ year (n)	*p*-value^**^
AST (IU/L)	30.48 ± 7.54 (1329)	27.59 ± 7.05^*^ (943)	26.01 ± 7.72^*^ (610)	24.89 ± 8.42^*^ (397)	23.71 ± 5.95^*^ (237)	22.95 ± 5.80^*^ (148)	<0.01
ALT (IU/L)	15.26 ± 7.25 (1328)	13.83 ± 6.65^*^ (943)	13.41 ± 6.97^*^ (610)	13.41 ± 7.30^*^ (396)	13.42 ± 5.14^*^ (237)	13.83 ± 7.74^*^ (148)	<0.01
Uric acid (mg/dL)	3.94 ± 0.87 (896)	4.28 ± 0.97^*^ (645)	4.40 ± 1.08^*^ (440)	4.55 ± 1.17^*^ (281)	4.75 ± 1.40^*^ (161)	4.83 ± 1.34^*^ (98)	<0.01
Total cholesterol (mg/dL)	173.24 ± 29.53 (1065)	166.80 ± 29.20^*^ (844)	162.81 ± 27.03^*^ (540)	162.56 ± 26.14^*^ (346)	163.03 ± 28.69^*^ (202)	158.73 ± 24.01^*^ (127)	<0.01
Triglyceride (mg/dL)	88.32 ± 55.55 (307)	100.66 ± 51.50^*^ (199)	96.73 ± 50.65 (100)	97.45 ± 52.10 (60)	111.41 ± 75.04 (37)	122.20 ± 77.49 (20)	0.01
Glucose (mg/dL)	92.08 ± 13.21 (1241)	98.40 ± 12.76^*^ (919)	97.75 ± 12.51^*^ (595)	97.43 ± 12.34^*^ (384)	97.95 ± 12.71^*^ (225)	98.66 ± 11.72^*^ (143)	<0.01
HbA1c (%)	5.28 ± 0.28 (204)	5.33 ± 0.28^*^ (554)	5.35 ± 0.28 (392)	5.34 ± 0.27^*^ (238)	5.36 ± 0.25 (150)	5.38 ± 0.25 (100)	<0.01

^*^p-value < 0.05 after GH treatment compared to baseline.

^**^p-value by analyzing generalized estimating equations over time.

Data are shown as the mean ± SD.

ALT, Alanine Aminotransferase; AST, Aspartate Aminotransferase; GH, Growth Hormone; GHD, Growth Hormone Deficiency; HbA1c, Glycosylated hemoglobin.

**Table 3B:** Changes in metabolic parameters after GH treatment in patients with SGA.

Variables	Baseline (n)	1^st^ year (n)	2^nd^ year (n)	3^rd^ year (n)	4^th^ year (n)	5^th^ year (n)	*p*-value^**^
AST (IU/L)	32.07 ± 7.53 (307)	28.78 ± 6.07^*^ (235)	27.34 ± 5.74^*^ (151)	26.49 ± 7.11^*^ (102)	25.57 ± 5.80^*^ (60)	24.28 ± 5.37^*^ (39)	<0.01
ALT (IU/L)	15.07 ± 6.26 (307)	14.34 ± 6.9 (235)	14.28 ± 6.63 (151)	14.67 ± 7.84 (102)	15.25 ± 6.66 (60)	15.46 ± 6.70 (39)	0.81
Uric acid (mg/dL)	3.92 ± 0.84 (208)	4.16 ± 0.89^*^ (153)	4.32 ± 1.03^*^ (104)	4.36 ± 0.74^*^ (62)	4.60 ± 1.37^*^ (32)	4.52 ± 1.34^*^ (19)	<0.01
Total cholesterol (mg/dL)	169.42 ± 27.45 (273)	164.36 ± 25.84^*^ (216)	162.63 ± 23.85^*^ (137)	164.94 ± 22.95^*^ (85)	168.05 ± 27.87 (46)	159.69 ± 20.98 (29)	<0.01
Triglyceride (mg/dL)	88.59 ± 57.73 (70)	92.44 ± 57.58 (62)	105.11 ± 58.90 (28)	120.00 ± 89.18 (17)	105.50 ± 63.32 (8)	107.50 ± 49.75 (6)	0.09
Glucose (mg/dL)	92.64 ± 13.49 (296)	97.03 ± 12.44^*^ (229)	98.71 ± 10.92^*^ (149)	97.07 ± 12.89^*^ (99)	96.92 ± 10.72^*^ (60)	100.53 ± 16.33 (38)	<0.01
HbA1c (%)	5.29 ± 0.22 (77)	5.29 ± 0.27 (136)	5.32 ± 0.24 (90)	5.31 ± 0.26 (70)	5.36 ± 0.26* (46)	5.37 ± 0.26^*^ (29)	0.01

^*^p-value < 0.05 after GH treatment compared to baseline.

^**^p-value by analyzing generalized estimating equations over time.

Data are shown as the mean ± SD.

ALT, Alanine Aminotransferase; AST, Aspartate Aminotransferase; GH, Growth Hormone; HbA1c, Glycosylated hemoglobin; SGA, Small for Gestational Age.

**Table 3C:** Changes in metabolic parameters after GH treatment in patients with GHD–SGA.

Variables	Baseline (n)	1^st^ year (n)	2^nd^ year (n)	3^rd^ year (n)	4^th^ year (n)	5^th^ year (n)	*p*-value^**^
AST (IU/L)	32.32 ± 7.93 (102)	29.58 ± 7.58^*^ (66)	27.35 ± 6.62^*^ (59)	25.17 ± 6.57^*^ (30)	24.39 ± 4.38^*^ (18)	27.30 ± 10.72^*^ (10)	<0.01
ALT (IU/L)	15.23 ± 5.48 (102)	14.03 ± 4.34 (66)	13.59 ± 4.58 (59)	13.13 ± 5.90 (30)	14.56 ± 6.90 (18)	17.50 ± 9.49 (10)	0.22
Uric acid (mg/dL)	3.99 ± 0.90 (67)	4.24 ± 0.91^*^ (51)	4.59 ± 1.03^*^ (39)	4.50 ± 1.07^*^ (21)	4.21 ± 1.12 (9)	4.43 ± 1.44 (6)	<0.01
Total cholesterol (mg/dL)	167.29 ± 26.60 (85)	162.60 ± 28.70 (59)	159.15 ± 28.78^*^ (54)	151.19 ± 28.97^*^ (27)	156.75 ± 27.40 (15)	159.29 ± 38.02 (9)	<0.01
Triglyceride (mg/dL)	86.54 ± 40.87 (24)	90.47 ± 38.22 (17)	89.60 ± 26.49 (10)	94.13 ± 36.66 (8)	83.00 ± 20.54 (4)	78.0 (1)	0.95
Glucose (mg/dL)	94.04 ± 12.87 (93)	97.26 ± 11.09^*^ (72)	94.26 ± 10.33 (53)	96.50 ± 12.75 (28)	93.69 ± 12.78 (16)	98.00 ± 11.89 (7)	0.36
HbA1c (%)	5.32 ± 0.20 (19)	5.33 ± 0.27 (40)	5.30 ± 0.20 (35)	5.31 ± 0.32 (18)	5.36 ± 0.27 (9)	5.41 ± 0.27 (7)	0.66

^*^p-value < 0.05 after GH treatment compared to baseline.

^**^p-value by analyzing generalized estimating equations over time.

Data are shown as the mean ± SD.

ALT, Alanine Aminotransferase; AST, Aspartate Aminotransferase; GH, Growth Hormone; HbA1c, Glycosylated hemoglobin; SGA, Small for Gestational Age.

The baseline total cholesterol level was 173.24 ± 29.53 mg/dL, which progressively decreased during GH treatment, with statistically significant reductions observed (p < 0.01 for all, **Figure 3D**). In contrast, TG levels did not exhibit significant changes throughout the treatment period compared to baseline levels, except for a transient reduction in the first year after GH treatment. However, GEE analysis indicated a tendency for TG levels to increase over time (p = 0.01). Random blood glucose levels increased during the first of GH treatment (**Figure 3E**); however, all values remained within the normal range. Similarly, HbA1c levels revealed significant changes in the first and third years of GH treatment (p < 0.01 for all), yet all values remained within the normal range (**Figure 3F**).

The pattern of metabolic parameter changes in the SGA group was similar to that observed in the GHD group. AST and ALT levels significantly decreased to within the normal range. Uric acid levels demonstrated a continuous increase throughout the treatment period but remained within the normal range (p < 0.01 in the first, second, third, and fourth years; p = 0.02 in the fifth year). Total cholesterol levels significantly decreased over the 5-year treatment period compared to baseline (p < 0.01 for all years), whereas TG levels did not exhibit significant changes. Conversely, glucose levels exhibited the most pronounced increase in the first year of GH treatment (p < 0.01), followed by a relatively stable pattern thereafter. However, HbA1c levels did not significantly change before and after GH treatment, although GEE analysis indicated a slight increase (p < 0.01), with values remaining within the normal range.

In patients with GHD and SGA, AST levels significantly improved relative to baseline values after the initiation of GH treatment (all, p < 0.01). Uric acid concentrations significantly increased during the first, second, and third years of treatment (all p < 0.01), while total cholesterol levels significantly declined in years 2 and 3. Conversely, fasting glucose levels increased significantly only during the first year of therapy. GEE modeling demonstrated a significant time-dependent decrease in AST and total cholesterol levels (both, p < 0.01), alongside a significant time-dependent increase in uric acid levels (p < 0.01).

There were minimal significant differences in metabolic parameter changes between the GHD and SGA groups (**Table 3C**). However, the increase in uric acid levels was significantly greater in the GHD group than in the SGA group in the second year (p = 0.02) and in the third year (p = 0.02) (**Figure 3C**). Additionally, glucose levels significantly increased in both groups after the first year of GH treatment; however, the increase was more pronounced in the GHD group than in the SGA group (p = 0.048) (**Figure 3E**).

## 4 Discussion

This study demonstrates that long-term GH treatment in patients with GHD and SGA is effective in improving metabolic parameters, particularly BMI, hepatic indices, and lipid profiles, over a 5-year follow-up period and also promotes linear growth. Our findings align with those of previous studies (19, 20), indicating that GH treatment leads to a substantial increase in height SDS in both groups, with a more pronounced response over time in patients experiencing hormone deficiencies or restricted fetal development. The greatest height SDS increase occurred in the first year of treatment, reaching 0.78 ± 0.38 (GHD: 0.78 ± 0.37; SGA: 0.79 ± 0.31; GHD and SGA: 0.80 ± 0.44), which is consistent with the results of previous studies reporting a first-year increment of 0.72 SDS. After 5 years of treatment, the total height SDS gain was 1.78 ± 0.94, 1.94 ± 0.82, and 1.64 ± 0.85 in the GHD, SGA, and GHD and SGA groups, aligning with the findings of other long-term observational cohort studies. For example, the Kabi/Pfizer International Growth Database (KIGS) cohort study (21, 22) reported median near-adult height (NAH)-SDS gains of 1.79 in boys and 2.07 in girls with idiopathic GHD. In the SGA group, the reported gains were 1.34 and 1.57 in boys and girls, respectively. However, in this study, the greater height gain observed in the SGA group after 5 years may be partly explained by the smaller number of patients who completed the full 5-year follow-up in this subgroup.

Additionally, the weight SDS exhibited significant improvements at all time points, although the BMI trends varied over different periods. The initial decline in BMI SDS during the first year of treatment may be attributed to the rapid increase in height, resulting in a relative reduction in BMI. However, BMI gradually increased over time, approaching the average range as treatment continued. Similar trends have been reported in other studies (15, 21), where BMI did not consistently increase in either the GHD or SGA groups. Notably, in this study, the SGA group demonstrated a greater improvement in BMI at years 3, 4, and 5 than at pre-treatment levels, which suggested that GH treatment may have a more pronounced effect on body composition in this subgroup. However, in the combined GHD and SGA group, BMI significantly increased only during the first year of GH treatment and showed no further significant change thereafter. The absence of sustained significance is likely attributable to the limited statistical power arising from the relatively small subgroup size. Moreover, compared with that in the SGA-only group, the magnitude of BMI improvement in the GHD and SGA groups was small, suggesting that the metabolic response to GH treatment may differ according to the underlying growth disorder.

Beyond physical development, biochemical markers provide additional insights into the metabolic impact of the intervention. In all groups, the liver function parameters, AST and ALT, exhibited significant reduction over the treatment period, reaching normal levels. This finding supports previous evidence suggesting that GH may contribute to improved hepatic function (23, 24). These findings are consistent with those of prior research highlighting the role of GH and insulin-like growth factor 1 (IGF-1) in reducing hepatic inflammation, oxidative stress, and lipid accumulation, which are key contributors to liver dysfunction (25). As the liver is a primary target organ for GH and IGF-1, patients with GHD are at an increased risk of developing MASLD; both GHD and MASLD are recognized as major complications in adults with GHD (8). Additionally, rapid postnatal weight gain in children with SGA is associated with an increased risk of MASLD (26). The findings of this study suggest that appropriate GH treatment may help reduce complications such as MASLD, underscoring its potential role in metabolic health management.

Uric acid levels gradually increased during GH treatment in all three groups. This metabolic change has been observed in patients with idiopathic short stature, suggesting that it may be a common response to GH treatment (27). The increase in uric acid levels during GH treatment is likely due to enhanced purine metabolism driven by increased protein turnover and muscle mass expansion. GH stimulates anabolic processes, including muscle growth and tissue remodeling, which elevates nucleotide turnover and subsequently increases uric acid production (28). Additionally, GH influences renal function by modulating uric acid excretion, potentially contributing to its accumulation. Despite this increase, uric acid levels remained within the normal range, suggesting physiological adaptation rather than a pathological concern. Regular metabolic monitoring is recommended to ensure that uric acid levels do not exceed normal limits, particularly in patients with metabolic risk factors. In the present study, the greater increase in uric acid levels in the GHD group than in the SGA group may be attributed to a larger height increment. To further support this mechanistic interpretation, we conducted a correlation analysis and found that changes in uric acid levels were positively associated with BMI SDS during the first three years of treatment and with IGF-1 SDS across all time points in the GHD group, suggesting that uric acid elevation reflects GH-induced anabolic activity rather than adverse metabolic disturbance.

Lipid metabolism is another key area of interest, with total cholesterol levels exhibiting a downward trend. This observation aligns with previous findings (15, 29) suggesting that GH treatment may positively influence lipid profiles by affecting fat distribution and metabolic function in both the GHD and GHD–SGA groups (30). Another study reported a reduction in total cholesterol levels after GH treatment, along with an increase in high-density lipoprotein cholesterol levels (31). However, some studies have demonstrated no significant improvement in lipid profiles before and after treatment, possibly due to small sample sizes limiting statistical significance. Experimental work in healthy volunteers shows that a brief increase in GH activity intensifies adipose lipolysis, driving a ~25% rise in hepatic VLDL-TG export, thereby supplying circulation with additional triglyceride-rich particles (32). This substrate-driven secretion, together with the transient insulin resistance and reduced peripheral lipoprotein lipase activity observed during early GH exposure, offers a plausible biological explanation for the modest, non-significant TG elevation documented in the present study. Notably, the differences between the two study groups were minimal, indicating the broad applicability of these findings across various etiologies of growth impairment. However, because several lipid-related parameters were obtained in the non-fasting state, postprandial variability might have contributed to the modest and inconsistent changes observed, especially in TG levels.

Markers of glucose regulation exhibited transient elevation, particularly within the first year of treatment, but remained within the normal range. Consistent with earlier studies, GH acts as an insulin-counterregulatory hormone (33), increases insulin resistance, and can consequently affect glucose metabolism in both GHD and SGA. HbA1c levels increased slightly over the treatment period, with significant changes noted in the first and third years.; however, these changes remained within clinically acceptable limits. This suggests that although glucose metabolism is influenced by treatment (4), it does not necessarily predispose individuals to long-term dysregulation (34). Notably, despite previous reports indicating an increased risk of metabolic disorders, such as insulin resistance and diabetes, in the SGA group (13), no significant changes in HbA1c levels were observed. This indicates minimal adverse effects of GH treatment. However, continuous monitoring of glucose-related indices remains essential, particularly in populations predisposed to metabolic disturbances.

Although most metabolic indices remained within normal limits throughout the 5-year treatment period, the clinical relevance of these findings should be interpreted in light of established pediatric thresholds. In this study, none of the group means for glucose, triglycerides, uric acid, or liver enzymes exceeded conventional reference limits (e.g., AST or ALT > 40 U/L, uric acid > 6.0 mg/dL, Total cholesterol > 200mg/dL, TG > 150 mg/dL, glucose > 100 mg/dL). These results suggest that GH treatment exerts mild, physiological metabolic shifts rather than pathological changes. Nevertheless, we recommend periodic metabolic assessment ideally every 6–12 months with particular attention to glucose regulation, including fasting glucose and HbA1c, as GH treatment can transiently influence insulin sensitivity. Regular monitoring of uric acid is also advised, especially in patients with obesity, SGA, or other metabolic risk factors.

This study had some limitations. First, as this was a retrospective study, the number of available metabolic parameter measurements was limited, and missing data were sporadic, with differences in sample sizes among the GHD, SGA, and GHD–SGA subgroups. However, despite these limitations, the 5-year follow-up period and multicenter cohort design provide substantial value. Second, non-fasting blood samples were used for several metabolic indices, including glucose, TG, and cholesterol. This approach increased the sample size and enabled longitudinal comparisons, but may have introduced potential bias due to postprandial variability. Because fasting data were not consistently collected across all centers, a sensitivity analysis limited to fasting samples could not be performed. Future studies that focus exclusively on fasting levels may yield more precise metabolic insights.

In conclusion, this study highlights that hormonal intervention in pediatric patients with growth impairment is effective not only for promoting linear growth but also for optimizing metabolic health. The observed reduction in hepatic enzyme levels and improvement in lipid profiles suggest broader systemic benefits beyond height gains. However, careful monitoring of glucose metabolism and uric acid levels is essential to ensure the long-term safety of this intervention. Future research should focus on strategies to minimize potential metabolic risks while maximizing the positive effects of GH treatment.

## Data Availability

The raw data supporting the conclusions of this article will be made available by the authors, without undue reservation.
