# Supplementary material for: Metabolic changes after growth hormone treatment in children with growth hormone deficiency and small for gestational age: LG observational study
Source: Front Endocrinol (Lausanne). 2025 Nov 19;16:1661018. doi: 10.3389/fendo.2025.1661018 (PMC12672343; doi:10.3389/fendo.2025.1661018)
Supplement: Supplementary file 1 [file Table1.docx]

|  | GHD group | SGA group | GHD–SGA group |
| --- | --- | --- | --- |
| 1^st^ year |  |  |  |
| N | 623 | 149 | 49 |
| *correlation coefficient* | 0.25 | 0.20 | 0.29 |
| p-value | **<0.01**^†^ | **0.01**^†^ | **0.04**^†^ |
| 2 ^nd^ year |  |  |  |
| N | 410 | 99 | 35 |
| *correlation coefficient* | 0.41 | 0.25 | 0.23 |
| p-value | **<0.01**^†^ | **0.01**^†^ | 0.18^†^ |
| 3 ^rd^ year |  |  |  |
| N | 267 | 56 | 20 |
| *Correlation coefficient* | 0.46 | 0.08 | 0.34 |
| p-value | **<0.01**^†^ | 0.54^†^ | 0.15^†^ |
| 4 ^th^ year |  |  |  |
| N | 153 | 32 | 8 |
| *Correlation coefficient* | 0.48 | 0.13 | 0.86 |
| p-value | **<0.01**^†^ | 0.49^†^ | **0.07**^†^ |
| 5 ^th^ year |  |  |  |
| N | 94 | 18 | 6 |
| *Correlation coefficient* | 0.36 | 0.67 | -0.32 |
| p-value | **0.04**^†^ | **0.03**^††^ | 0.54^††^ |

Supplementary 1. Correlation analysis between serum uric acid levels and BMI

^†^p-values were calculated by Spearman correlation

^††^p-values were calculated by Pearson correlation

GHD, Growth Hormone Deficiency; SGA, Small for Gestational Age; BMI, Body Mass Index

|  | GHD group | SGA group | GHD–SGA group |
| --- | --- | --- | --- |
| 1^st^ year |  |  |  |
| N | 623 | 149 | 49 |
| *correlation coefficient* | 0.12 | 0.11 | 0.09 |
| p-value | **<0.01**^†^ | 0.18^†^ | 0.54^††^ |
| 2 ^nd^ year |  |  |  |
| N | 410 | 99 | 35 |
| *correlation coefficient* | 0.21 | 0.16 | 0.25 |
| p-value | **<0.01**^†^ | 0.12^†^ | 0.15^††^ |
| 3 ^rd^ year |  |  |  |
| N | 267 | 56 | 20 |
| *correlation coefficient* | 0.26 | 0.02 | 0.09 |
| p-value | **<0.01**^†^ | 0.90^†^ | 0.70^†^ |
| 4 ^th^ year |  |  |  |
| N | 153 | 32 | 8 |
| *correlation coefficient* | 0.14 | 0.28 | 0.91 |
| p-value | 0.09^†^ | 0.13^††^ | **<0.01**^††^ |
| 5 ^th^ year |  |  |  |
| N | 94 | 18 | 6 |
| *correlation coefficient* | 0.15 | 0.62 | -0.30 |
| p-value | 0.16^†^ | **<0.01**^††^ | 0.57^††^ |

Supplementary 2. Correlation analysis between serum uric acid levels and BMI SDS

^†^p-values were calculated by Spearman correlation

^††^p-values were calculated by Pearson correlation

GHD, Growth Hormone Deficiency; SGA, Small for Gestational Age; BMI, Body Mass Index; SDS, Standard Deviation Score

Supplementary 3. Correlation analysis between serum uric acid levels and IGF-1 levels

|  | GHD group | SGA group | GHD–SGA group |
| --- | --- | --- | --- |
| 1^st^ year |  |  |  |
| N | 615 | 152 | 49 |
| *correlation coefficient* | 0.25 | 0.14 | 0.41 |
| p-value | **<0.01**^†^ | 0.09^†^ | **<0.01**^†^ |
| 2 ^nd^ year |  |  |  |
| N | 413 | 99 | 37 |
| *correlation coefficient* | 0.33 | 0.28 | 0.22 |
| p-value | **<0.01**^†^ | **<0.01**^†^ | 0.18^†^ |
| 3 ^rd^ year |  |  |  |
| N | 270 | 57 | 19 |
| *correlation coefficient* | 0.38 | -0.08 | 0.46 |
| p-value | **<0.01**^†^ | 0.53^†^ | **0.048**^†^ |
| 4 ^th^ year |  |  |  |
| N | 149 | 29 | 7 |
| *correlation coefficient* | 0.36 | 0.24 | 0.43 |
| p-value | **<0.01**^†^ | 0.21^††^ | 0.34^†^ |
| 5 ^th^ year |  |  |  |
| N | 91 | 19 | 6 |
| *correlation coefficient* | 0.33 | 0.27 | -0.26 |
| p-value | **<0.01**^†^ | 0.26^††^ | 0.62^††^ |

^†^p-values were calculated by Spearman correlation

^††^p-values were calculated by Pearson correlation

GHD, Growth Hormone Deficiency; SGA, Small for Gestational Age; IGF-1, Insulin like Growth Factor-1
